# Supplementary material for: Memory recall involves a transient break in excitatory-inhibitory balance
Source: eLife. 2021 Oct 8;10:e70071. doi: 10.7554/eLife.70071 (PMC8516417; doi:10.7554/eLife.70071)
Supplement: Supplementary file 1. — Using a general linear model (GLM), differences in sex (male or female) were regressed onto behavioural performance during both the inference test and associative test, and onto glu/GABA ratio during the question period of the inference test. No significant effect of sex was observed. [file elife-70071-supp1.docx]

**Supplementary File 1 | The effect of sex on behaviour and on glu/GABA ratio in V1**

| **Test** | **T-statistic** | **p-value** |
| --- | --- | --- |
| Behavioural performance during the *inference test*  (performed inside MRI scanner) | t_17_=0.50 | p=0.622 |
| Behavioural performance during the post-scan *associative test* | t_17_=0.42 | p=0.674 |
| glu/GABA ratio in V1 during the question period in the *inference test* | t_16_=1.67 | p=0.215 |
