# Supplementary material for: Memory recall involves a transient break in excitatory-inhibitory balance
Source: eLife. 2021 Oct 8;10:e70071. doi: 10.7554/eLife.70071 (PMC8516417; doi:10.7554/eLife.70071)
Supplement: Supplementary file 2. — The number of trials per condition, reported as mean ± SEM. [file elife-70071-supp2.docx]

**Supplementary File 2 | Number of trials per condition**

| **Categorization criteria** | **No. of trials per condition** | |
| --- | --- | --- |
|  | **‘Remembered’** | **‘Forgotten’** |
| Using performance on both the inference test and post-scan associative test: ‘correctly inferred & recalled’ vs ‘incorrectly inferred \| not recalled’ (see Figure 3B) | 38.95 ± 1.56 | 39.32 ± 1.61 |
| Using performance on inference test alone: ‘correctly inferred’ vs 'incorrectly inferred’ | 59.74 ± 1.07 | 18.53 ± 1.04 |
| Using performance on post-scan associative test alone: ‘recalled’ vs ‘not recalled’ | 42.84 ±1.46 | 25.53 ±1.51 |
