# Supplementary material for: Memory recall involves a transient break in excitatory-inhibitory balance
Source: eLife. 2021 Oct 8;10:e70071. doi: 10.7554/eLife.70071 (PMC8516417; doi:10.7554/eLife.70071)
Supplement: Supplementary file 3. — The number of inference trials per condition (‘remembered’ and ‘forgotten’, see Materials and methods for definition), split according to whether the auditory cue was indirectly associated with a rewarding or neutral outcome (set 1: rewarded; set 2: neutral), reported as mean ± SEM. There was no significant difference in the number of trials split by set (memory set, two-way ANOVA: F(1,68)=0.67, p = 0.424). Notably, the average difference in the total number of trials in set 1 and 2 was less than one trial, suggesting memory recall was not confounded by reward status. [file elife-70071-supp3.docx]

**Supplementary File 3 | Number of trials split according to outcome**

| **Condition** | **No. of trials in inference test** | |
| --- | --- | --- |
|  | **Set 1 (rewarded)** | **Set 2 (neutral)** |
| ‘Remembered’ | 18.0 ± 1.15 | 17.33 ± 0.91 |
| ‘Forgotten’ | 15.83 ± 0.89 | 16.72 ± 0.90 |
