# Supplementary material for: Memory recall involves a transient break in excitatory-inhibitory balance
Source: eLife. 2021 Oct 8;10:e70071. doi: 10.7554/eLife.70071 (PMC8516417; doi:10.7554/eLife.70071)
Supplement: Supplementary file 4. — The fMRI blood oxygen level-dependent (BOLD) signal was assessed for a contrast comparing ‘remembered’ and ‘forgotten’ trials (Figure 3B) in the inference test. Brain regions that survived whole-volume correction for multiple comparisons are listed (p < 0.05 with whole-brain family wise error [FWE] correction at the cluster level). Montreal Neurological Institute (MNI) coordinates are listed. Notably, the activation in hippocampus was bilateral but the peak on the right-hand side was not significant when applying whole-brain FWE correction (t17 = 3.74, p > 0.05 [x = 22, y=-24, z=−13]). [file elife-70071-supp4.docx]

**Supplementary File 4 | fMRI contrast for ‘remembered’ – ‘forgotten’**

| Brain region | P _FWE-corr, cluster level_ | T | Peak coordinate in cluster | | |
| --- | --- | --- | --- | --- | --- |
|  |  |  | **x** | **y** | **z** |
| Left hippocampus | P=0.017 | 4.33 | -18 | -28 | -8 |
| Visual cortex | P<0.001 | 5.92 | 4 | -78 | 0 |
| Right auditory cortex | P<0.001 | 5.58 | 38 | -26 | 16 |
| Left posterior parietal cortex | P<0.001 | 5.00 | -44 | -52 | 22 |
