# Supplementary material for: Memory recall involves a transient break in excitatory-inhibitory balance
Source: eLife. 2021 Oct 8;10:e70071. doi: 10.7554/eLife.70071 (PMC8516417; doi:10.7554/eLife.70071)
Supplement: Supplementary file 5. — Inter-subject covariances (%) for the key metabolite measurements during the ‘Question’ period of inference trials (presented in Figure 4D–E). [file elife-70071-supp5.docx]

**Supplementary File 5 | Inter-subject covariance of glutamate and GABA**

|  | glutamate | GABA |
| --- | --- | --- |
| ‘Remembered’ | 6.71 | 33.25 |
| ‘Forgotten’ | 5.69 | 31.04 |
