# Supplementary material for: Memory recall involves a transient break in excitatory-inhibitory balance
Source: eLife. 2021 Oct 8;10:e70071. doi: 10.7554/eLife.70071 (PMC8516417; doi:10.7554/eLife.70071)
Supplement: Supplementary file 6. — The number of spectra contributing to metabolite estimates during the various trial periods in the inference test (mean ± SEM). [file elife-70071-supp6.docx]

**Supplementary File 6 | Average number of spectra (NEX)**

|  | Tone | Question | ITI |
| --- | --- | --- | --- |
| ‘Remembered’ | 60.05±2.73 | 16.17±1.08 | 35.53±1.76 |
| ‘Forgotten’ | 47.42±2.23 | 14.61±1.21 | 28.37±1.49 |
