# Supplementary material for: Memory recall involves a transient break in excitatory-inhibitory balance
Source: eLife. 2021 Oct 8;10:e70071. doi: 10.7554/eLife.70071 (PMC8516417; doi:10.7554/eLife.70071)
Supplement: Supplementary file 7. — The relationship between functional magnetic resonance imaging (fMRI) and fMRS during ‘remembered’ versus ‘forgotten’ trials in the inference test was assessed. To this end, fMRS measures of glu/GABA ratio from primary visual cortex (V1) for ‘remembered’–‘forgotten’ were included as covariates in a group analysis for the equivalent fMRI contrast (p < 0.05 with family wise error [FWE] correction at the cluster level). The only brain region to survive whole-brain correction for multiple comparisons was the left hippocampus. Thus, the BOLD signal in left hippocampus significantly predicted individual differences in glu/GABA ratio measured from V1 during memory recall; MNI coordinates. [file elife-70071-supp7.docx]

**Supplementary File 7 | Covariance between hippocampal BOLD signal and fMRS for remembered vs. forgotten**

| Brain region | P _FWE-corr, peak level_ | T | Coordinate | | |
| --- | --- | --- | --- | --- | --- |
|  |  |  | **x** | **y** | **z** |
| Left hippocampus | P=0.005 | 11.37 | -26 | -12 | -16 |
