# Supplementary material for: Memory recall involves a transient break in excitatory-inhibitory balance
Source: eLife. 2021 Oct 8;10:e70071. doi: 10.7554/eLife.70071 (PMC8516417; doi:10.7554/eLife.70071)
Supplement: Supplementary file 8. [file elife-70071-supp8.docx]

**Supplementary File 8 |** **Minimum Reporting Standards in MRS (MRSinMRS) checklist**

| Site (Name or Number) | FMRIB, Wellcome Centre for Integrative Neuroimaging (University of Oxford) |
| --- | --- |
| 1. Hardware |  |
| a. Field strength [T] | 7 |
| b. Manufacturer | Siemens |
| c. Model (software version if available) | Magnetom 7T (VB17a) |
| d. RF coils: nuclei (transmit/ receive), number of channels, type, body part | 1-channel transmit and a 32-channel receive phased-array head coil (Nova Medical Inc., USA) |
| e. Additional hardware | Two 110 × 110 × 5 mm^3^ Barium Titanate dielectric pads (4:1 ratio of BaTiO_3_:D_2_O, relative permittivity around 300) over occipital lobe |
| 2. Acquisition |  |
| a. Pulse sequence | combined fMRI-fMRS sequence fMRI: 3D EPI fMRS: semi-LASER |
| b. Volume of Interest (VOI) locations | Visual cortex |
| c. Nominal VOI size [cm^3^, mm^3^] | 2 × 2 × 2 cm^3^ |
| d. Repetition Time (TR), Echo Time (TE) [ms, s] | TR = 4105 ms TE = 36 ms |
| e. Total number of Excitations or acquisitions per spectrum  In time series for kinetic studies   1. Number of Averaged spectra (NA) per time-point 2. Averaging method (e.g. block-wise or moving average) 3. Total number of spectra (acquired / in time-series) | Average total number of spectra acquired per participant: N=457 (SD: 35.62)  Between 8 and 81 spectra per condition (see Supplementary File 6). Participants with less than 8 spectra per condition (e.g. ‘remembered’ or ‘forgotten’) were not included in the analysis.  Spectra were averaged according to condition (‘remembered’/‘forgotten’). |
| f. Additional sequence parameters  (spectral width in Hz, number of spectral points, frequency offsets)  If STEAM:, Mixing Time (TM)  If MRSI: 2D or 3D, FOV in all directions, matrix size, acceleration factors, sampling method | Spectral width: 6 kHz Spectral points: 2048 frequency offset: -2.0 ppm |
| g. Water Suppression Method | VAPOR |
| h. Shimming Method, reference peak, and thresholds for “acceptance of shim” chosen | 3D head shim (GRESHIM) followed by FASTESTMAP, threshold for acceptable shim was linewidth water <16 Hz |
| i. Triggering or motion correction method  (respiratory, peripheral, cardiac triggering, incl. device used and delays) | N/A |
| 3. Data analysis methods and outputs |  |
| a. Analysis software | LCmodel (fitting and quantification, version 6.3-1B), MRspa (preprocessing, version 1.4) |
| b. Processing steps deviating from quoted reference or product | Residual water component was removed using Hankel Lanczos Singular Value Decomposition (HLSVD). |
| c. Output measure  (e.g. absolute concentration, institutional units, ratio)Processing steps deviating from quoted reference or product | Concentrations relative to total Creatine. |
| d. Quantification references and assumptions, fitting model assumptions | The basis set included simulated model spectra of alanine, aspartate, ascorbate/vitamin C, glycerophosphocholine, phosphocholine, creatine, phosphocreatine, GABA, glucose, glutamine, glutamate, glutathione, myo-inositol, Lactate, N-acetylaspartate, N-acetylaspartylglutamate, phosphoethanolamine, scyllo-inositol, taurine and experimentally measured macromolecules. Basis set is made available on GitHub.  Prior constraints that fix GABA estimates relative to more abundant neurochemicals were turned off (i.e. NRATIO set to 0). |
| 4. Data Quality |  |
| a. Reported variables  (SNR, Linewidth (with reference peaks)) | Across all spectra (Figure 4–figure supplement 3):  SNR: 51.1± 2.37% (mean ± SEM) FWHM from LCModel: 9.86±0.30 Hz (mean ± SEM) Total Creatine Linewidth: 11.01±0.32Hz (mean ± SEM) |
| b. Data exclusion criteria | Subjects were excluded from an analysis if there were less than 8 spectra acquired for one or more of the conditions of interest (n=1 subject excluded for analyses concerning the question period due to less than 8 spectra in the ‘forgotten’ condition). |
| c. Quality measures of postprocessing Model fitting (e.g. CRLB, goodness of fit, SD of residual) | Across all spectra (Figure 4–figure supplement 3, mean ± SEM)):  CRLB glutamate: 4.42±0.43%  CRLB GABA: 11.37±0.94% Intra-subject covariance glutamate: 2.68±0.62% Intra-subject covariance GABA was 8.74±1.95%  Inter-subject covariance (Supplementary File 4):  glutamate: 5.69-6.71% GABA: 31.04-33.25% |
| d. Sample Spectrum | Figure 3E |
